# Earthquake victims in focus: a cross-sectional examination of trauma and management in intensive care unit

**DOI:** 10.1186/s12873-024-00949-4

**Published:** 2024-02-20

**Authors:** Kaniye Aydin, Aysun Ozel Yesilyurt, Ferhat Cetinkaya, Mehmet Gokhan Gok, Omer Dogan, Dilek Ozcengiz

**Affiliations:** 1 Division of Medical Intensive Care Unit, Department of Internal Medicine, School of Medicine, Cukurova University, Adana, Türkiye; 2 Department of Anesthesiology and Reanimation, School of Medicine, Cukurova University, Adana, Türkiye

**Keywords:** Earthquake, Victims, Intensive care unit, Mortality, CRAMS score, Intra-abdominal pressure

## Abstract

**Background:**

After the Kahramanmaras earthquake of February 6, 2023, the disaster of the century, a significant number of victims were admitted to intensive care units (ICUs). In this study, we aimed to share the characteristics and management of critical earthquake victims and shed light on our experiences as intensivists in future earthquakes.

**Methods:**

The study included 62 earthquake victims in two tertiary ICUs. Demographic characteristics, laboratory findings, clinical characteristics, trauma and disease severity scores, treatments administered to patients, and the clinical course of the patients were recorded retrospectively. The patients were divided into two groups, survivors and nonsurvivors, according to 7-day mortality and into two groups according to the duration of their stay under the rubble: those who remained under the rubble for 72 hours or less and those who remained under the rubble for more than 72 hours. A receiver operating characteristic (ROC) curve analysis was used to determine the best cutoff value for the ‘Circulation, Respiration, Abdomen, Motor, and Speech’ (CRAMS) score.

**Results:**

The median age of the 62 patients included in the study was 35.5 (23-53) years. The median length of stay under the rubble for the patients was 30.5 (12-64.5) hours. The patient was transferred to the ward with a maximum duration of 222 hours under the rubble. The limb (75.8%) was the most common location of trauma in patients admitted to the ICU. Crush syndrome developed in 96.8% of the patients. There was a positive correlation between the development of acute kidney injury (AKI) and myoglobin, serum lactate, and uric acid levels (*r* = 0.372, *p* = 0.003; *r* = 0.307, *p* = 0.016; *r* = 0.428, *p* = 0.001, respectively). The best cutoff of the CRAMS score to predict in-7-day mortality was < 4.5 with 0.94 area under the curve (AUC); application of this threshold resulted in 75% sensitivity and 96.3% specificity.

**Conclusion:**

Search and rescue operations should continue for at least ten days after an earthquake. The CRAMS score can be used to assess trauma severity and predict mortality in critically ill earthquake victims.

## Background

Earthquakes are sudden natural disasters that are common all over the world. On February 6, 2023, at 04:17 and 13:24 Turkiye time, two earthquakes of magnitude 7.7 Mw (focal depth=8.6 km) and 7.6 Mw (focal depth=7 km) on the Richter scale occurred in the Pazarcik and Elbistan districts of Kahramanmaras [1]. On February 20, 2023, at 20:04 Turkiye time, an earthquake with a magnitude of 6.4 Mw occurred, the epicenter of which was Hatay Yayladagi [1]. The earthquakes in question caused great destruction in 11 provinces in total. These earthquakes were unprecedented disasters in recent history in terms of intensity and area covered. In fact, as a result of earthquakes, more than 48 thousand people lost their lives [1]. During earthquakes, the most common cause of death is trauma. On the other hand, people who survive the rubble usually suffer multiple tissue and organ injuries as a result of trauma. The most important way to reduce mortality after these disasters is to know about traumatic complications, crush syndrome, and its treatments [2].

A significant portion of the survivors under the rubble needed hospitalization in the intensive care unit (ICU). In the literature, there are few studies showing the clinical course of earthquake survivors and the experience of clinicians in the ICU [3, 4]. Until now, there has been no detailed study showing the laboratory, clinical characteristics, course of critical earthquake victims, and the treatments applied. There have been earthquakes in our country and around the world thus far, and they will continue to happen. The aim of this study was to determine the clinical characteristics of earthquake survivors in the ICU, their clinical course, the treatments given to the patients, the complications seen in the patients, the management of complications, and the factors causing mortality and to share our clinical experience. Our experience will shed light on clinicians in future earthquakes.

## Materials and methods

### Study design

This retrospective, single-center, cross-sectional study was conducted with patients diagnosed with earthquake victims in the two ICUs at Cukurova University Faculty of Medicine Balcali Hospital. The local ethics committee approved the study protocol (Date: April 7, 2023; Reference Number: 22/132). The research was carried out in conformity with the ethical guidelines outlined in the Helsinki Declaration of 1964 and its subsequent amendments.

### Setting

The study was carried out in the two ICUs after it was approved by the local Clinical Research Ethics Committee. This retrospective study included patients ≥ 18 years old with patients diagnosed with earthquake victims who were followed in the two ICUs between February 6 and February 21, 2023. The demographic and clinical characteristics of the patients, treatments applied to the patients, clinical courses, and 7-day mortality were collected from the patient files and the hospital information management system.

### Participants

**Fig. 1:**
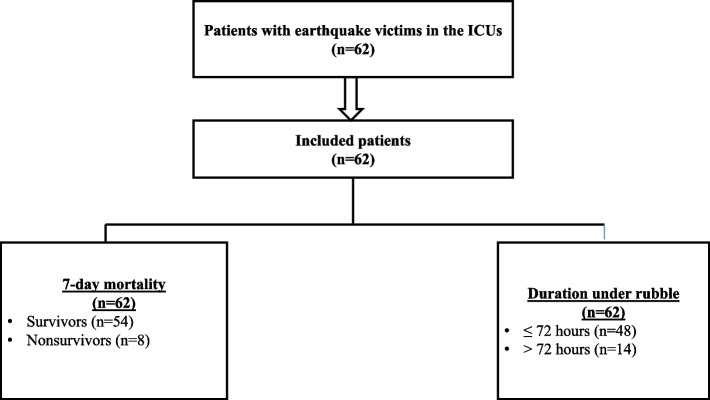
Flowchart of patients with earthquake victims in the ICUs

A total of 62 patients over 18 years of age who were earthquake victims in the ICUs met the inclusion criteria (Fig. 1). The exclusion criteria were patients under 18 years of age and nonearthquake victims. The patients were divided into two groups according to 7-day mortality: survivors and nonsurvivors. The patients were also divided into two groups according to the duration of their stay in the rubble: those who stayed 72 hours or less and those who stayed longer than 72 hours.

### Variables

In order to ensure the robustness and appropriateness of the study questionnaires, we convened an expert panel consisting of 2 intensivists, 3 internists, and 1 pulmonologist from the study team, along with 2 intensivists from outside the study. These experts were selected based on their extensive experience and expertise in the relevant field. The initial phase involved a comprehensive review of the existing literature, utilizing reputable databases such as PubMed, Cochrane Central, Web of Science, and Google Scholar. This literature review served as the foundation for identifying potential variables. Subsequently, the expert panel conducted a thorough examination of the identified parameters, assessing their relevance and significance to the research objectives. The variables that emerged from this scrutiny were then presented to the expert panel for approval. Any discrepancies or concerns were addressed during this process. The variables that had been selected as study questionnaires were approved collectively by the experts in the field. Ultimately, the variables selected as study questionnaires received collective approval from the assembled experts in the field, ensuring the validity and reliability of our chosen parameters.

The demographic characteristics of the patients (age, sex), the place of admission to the ICU (emergency department, inpatient service), comorbidities, the city in which the patient was trapped under the rubble, the duration from emergency service to ICU admission for all patients, and the duration of the patient's stay under the rubble were recorded on the study form. The Charlson comorbidity index (CCI), injury severity score (ISS), Glasgow coma scale (GCS) score, acute physiology and chronic health evaluation (APACHE) II score, sequential organ failure assessment (SOFA) score, ‘Circulation, Respiration, Abdomen, Motor, and Speech’ (CRAMS) score, modified nutrition risk in the critically ill (mNUTRIC) score, and revised trauma score (RTS) were calculated [5–8]. If the intra-abdominal pressures of patients were measured, they were recorded on the study form [9]. Vital findings, peripheral arterial oxygen saturation (SpO_2_) and laboratory results at admission to the ICUs were recorded on the form. Trauma sites (limb, thoracic, abdominal, head, pelvic, spinal); presence of trauma surgery, fracture, compartment syndrome, fasciotomy, and amputation; presence of pneumothorax, pneumomediastinum, hemothorax, or subcutaneous emphysema; whether a tube thoracostomy was performed; and whether trauma-related complications developed [acute kidney injury (AKI), crush syndrome, infection, sepsis, septic shock, deep vein thrombosis, disseminated intravascular coagulation, trauma-related coagulopathy] were recorded. The fluids given to the patients and the type of fluids, whether the patients needed blood transfusion (type of blood product), whether the patients needed vasopressors, inotropic agents, furosemide, intravenous nitroglycerin, anticoagulants, sedation, and analgesia, the type of oxygen treatments administered to the patients at admission to the ICUs and during the ICU stay, the 24-hour fluid volume and urine output of the patients, the need for renal replacement therapy (RRT), 7-day mortality, and the duration of hospital and ICU stay were analyzed.

### Statistical analysis

Continuous data are summarized as the mean and standard deviation or median and quartiles (Q1–Q3), and categorical variables are expressed as numbers and percentages. The patients were divided into two groups according to 7-day mortality: survivors and nonsurvivors. The patients were also divided into two groups according to the duration of their stay in the rubble: those who stayed 72 hours or less and those who stayed longer than 72 hours. To compare categorical variables between the groups, the chi-square or Fisher’s exact test was performed. The Kolmogorov-Smirnov test was used to confirm the normality of the distribution for continuous variables. Student's t test or the Mann-Whitney U test was performed to compare continuous variables between two groups. Variables significant at the *p*<0.25 level in the univariate analysis were included in the logistic regression analysis. In a multivariate analysis, logistic regression analysis was used to examine independent predictors of survival using potential factors identified in previous analyses. A receiver operating characteristic (ROC) curve analysis was performed to identify the optimal cutoff point for the CRAMS score. In the presence of a significant cutoff, the sensitivity, and specificity of these limits were calculated. Correlation coefficients and statistical significance were calculated with the Spearman test for relationships between variables that were ordinal or at least one of which was not normally distributed. All analyses were performed using IBM SPSS 20.0 (Armonk, NY: IBM Corp.). The statistical level of significance for all tests was set at 0.05.

## Results

The study involved 62 patients, and 28 (45.2%) of them were female (Table 1). The median age of the patients was 35.5 (23-53) years. Fifty-five (88.7%) of our patients came from Hatay, where the earthquake caused great damage. The median length of stay under the rubble for the patients was 30.5 (12-64.5) hours. The patient who remained under the rubble for the maximum time (222 hours) had thoracic trauma, brachial plexus injury and the highest observed serum sodium level (168 mmol/L); however, AKI was not observed in this patient. A weak positive correlation was observed between the duration of stay under the rubble and APACHE II and SOFA scores (*r*=0.257, *p*=0.044; *r*=0.263, *p*=0.039, respectively), but no correlation was observed with other scores. A high level of positive correlation was observed between the duration of exposure to rubble and serum sodium, and a moderate level of positive correlation was observed between the duration of exposure to rubble and blood urea nitrogen (BUN) level (*r* = 0.782, *p*<0.001; *r* = 0.667, *p*<0.001, respectively). A moderately positive correlation was observed between the duration of exposure to rubble and creatinine kinase (CK) levels (*r* = 0.394, *p* = 0.002). There was no correlation between the duration of exposure to rubble and AKI, the development of crush syndrome, or 7-day mortality. There was a moderately positive correlation between the duration of exposure to rubble and amputation (*r* = 0.432, *p*<0.001).

**Table 1:** Demographic characteristics of earthquake victims

**Characteristics**		**7-day mortality**			**Duration under the rubble**		
	**All patients (*n*=62)**	**Survivor (*n*=54)**	**Nonsurvivor (*n*=8)**	***p*-value**	**≤72 hours (*n*=48)**	**>72 hours (*n*=14)**	***p*- value**
**Median (Q1-Q3) or n (%)**							
**Age (years)**	35.5(23-53)	33(23-50.3)	52(34.3-71.3)	0.038	36.5(23-55.3)	33.5(28-49.3)	0.853
**Gender**							
Female	28(45.2)	25(46.3)	3(37.5)		22(45.8)	6(42.9)	1.000
Male	34(54.8)	29(53.7)	5(62.5)	0.719	26(54.2)	8(57.1)	
**Admission to ICU**							
From emergency service	45(72.6)	37(68.5)	8(100)	0.094	31(64.6)	14(100)	0.007
From inpatient service	17(27.4)	17(31.5)			17(35.4)		
**Duration from emergency service to ICU admission for all patients (hours)**	7.7(4.4-15.2)	7.7(4.4-16.1)	8(3-14.2)	0.809	10(4.6-21.7)	5(2.1-8)	0.012
**City**							
Hatay	55(88.7)	47(87)	8(100)	0.760	45(93.8)	10(71.4)	0.012
Kahramanmaras	4(6.5)	4(7.4)			1(2.1)	3(21.4)	
Adiyaman	1(1.6)	1(1.9)				1(7.1)	
Osmaniye	2(3.2)	2(3.7)			2(4.2)		
**Duration under the rubble (hours)**	30.5(12-64.5)	29(11.5-64.5)	41.5(30.5-78.3)	0.284			
**Comorbidities**							
Diabetes mellitus	4(6.5)	3(5.6)	1(12.5)		4(8.3)		
Hypertension	5(8.1)	3(5.6)	2(25)		4(8.3)	1(7.1)	
CAD&HF	5(8.1)	4(7.4)	1(12.5)		4(8.3)	1(7.1)	
Asthma	1(1.6)	1(1.9)				1(7.1)	
Malignancy	2(3.2)	1(1.9)	1(12.5)		1(2.1)	1(7.1)	
Chronic kidney disease	1(1.6)	1(1.9)				1(7.1)	
**CCI**	0(0-1)	0(0-1)	0(0-3.75)	0.270	0(0-1)	0(0-0.25)	0.508
**GCS score**	14(3-15)	14(10-15)	3(3-3)	<0.001	14(7-15)	11(3-114)	0.139
**APACHE II score**	20.1(15-29.3)	19.5(15-29)	28(23-37.8)	0.057	18(15-26.8)	30.5(20-39.3)	0.005
**Revised trauma score**	5.5(3-11)	8(3-12)	3(3-7.8)	0.099	8(3-12)	3.5(3-8.8)	0.223
**Injury severity score**	24.5(11.8-38)	27(11-38.8)	16.5(12-29.3)	0.429	22(11.3-38)	26.5(15-47.3)	0.600
**CRAMS score**	7.5(5-9)	8(6-9)	4(2.3-4.8)	<0.001	8(6-9)	6(4.5-9.3)	0.454
**SOFA score**	6(4-8)	6(4-6.5)	9(4.5-11.5)	0.027	5(4-6)	7(6-10.5)	0.010
**mNUTRIC score**	3(1.75-4)	3(1-4)	5(2-5.75)	0.012	3(2-4)	3(1-5)	0.726

*ICU* Intensive care unit, *CAD&HF* Coronary artery disease &heart failure, *CCI* Charlson comorbidity index, *GCS* Glasgow coma scale, *APACHE II* Acute Physiology and Chronic Health Evaluation, *CRAMS* Circulation, Respiration, Abdomen, Motor, and Speech, *SOFA* Sequential Organ Failure Assessment, *mNUTRIC* Modified Nutrition Risk in the Critically Ill

The CRAMS score was clinically and significantly lower in the nonsurvivors than in the survivors (*p*<0.001). The SOFA and mNUTRIC scores were clinically and significantly higher in the nonsurvivors than in the survivors (*p*<0.05). There was a moderately positive correlation between the ISS and the need for RRT (*r*=0.321, *p*=0.001). There was a negative correlation between the CRAMS score and the development of AKI and crush syndrome (*r* = -0.387, *p* = 0.002; r = -0.278, p = 0.028, respectively). There was a weak positive correlation between the mNUTRIC score and the development of crush syndrome (*r* = 0.290, *p* = 0.022).

Table 2 shows the vital signs of earthquake victims upon ICU admission. The nonsurvivors were hypothermic compared to the survivors (*p*<0.05). Systolic, diastolic, and mean blood pressures and SpO_2_ were statistically and clinically significantly lower in nonsurvivors than in survivors (*p*<0.05). Table 3 demonstrates the laboratory results of earthquake victims on ICU admissions. Blood gas pH and bicarbonate levels were significantly lower and lactate levels were higher in nonsurvivors than in survivors (*p*<0.05).

**Table 2:** The vital signs of earthquake victims

**Characteristics**		**7-day mortality**			**Duration under the rubble**		
	**All patients (*n*=62)**	**Survivor (*n*=54)**	**Nonsurvivor (*n*=8)**	***p*-value**	**≤72 hours (*n*=48)**	**>72 hours (*n*=14)**	***p*- value**
**Median (Q1-Q3) or Mean±SD**							
**Fever (**^**0**^**C)**	36.4(36-36.8)	36.6(36-36.9)	35.8(35.1-36)	0.005	36.5(36-37)	36.3(36-36.7)	0.378
**Heart rate (beats/min)**	115.9±18.1	115.9±17.4	115.8±23.7	0.975	117.6±17.5	110.1±19.4	0.175
**Respiratory rate (breaths/min)**	22.4±4.9	22.4±4.7	22.5±6.3	0.953	23.2±4.4	19.7±5.6	0.047
**Systolic blood pressure (mmHg)**	120(105.8-137)	120(110-137)	99.5(90-112)	0.008	120(107.8-137)	120(99-133.8)	0.667
**Diastolic blood pressure (mmHg)**	68.5±15.2	70.4±14.2	55.5±16.1	0.008	68.1±15.3	69.7±15.2	0.726
**Mean arterial pressure (mmHg)**	84.9±15.5	87.2±14.5	69.4±14.3	0.002	85.1±16.1	84.2±14.2	0.856
**SpO**_**2**_** (%)**	98(92.8-100)	98(94-100)	87.5(83.5-97.5)	0.010	95.5(92-98)	98.5(97.8-100)	0.016

*SpO*_*2*_ Peripheral arterial oxygen saturation

**Table 3:** Laboratory results of earthquake victims

**Characteristics**		**7-day mortality**			**Duration under the rubble**		
	**All patients (*n*=62)**	**Survivor (*n*=54)**	**Nonsurvivor (*n*=8)**	***p*-value**	**≤72 hours (*n*=48)**	**>72 hours (*n*=14)**	***p*- value**
**Median (Q1-Q3), mean**±SD** or n (%)**							
**White blood cell (10**^**3**^**/μL)**	18.9(13.8-28.5)	18.9(14.2-28.5)	15.1(7.8-33.0)	0.549	18.9(12.8-30.5)	18.9(14.3-24.2)	0.906
**Hemoglobin (g/dL)**	12.8±3.2	12.9±3.2	11.6±3.5	0.253	12.9±3.3	12.4±2.9	0.587
**Platelet (10**^**3**^**/μL)**	213(152.5-256.3)	215.5(159.8-256.3)	188(92.8-261.5)	0.339	215.5(164-262.3)	183(117.8-243)	0.213
**pH**	7.28(7.2-7.36)	7.3(7.25-7.36)	7(7-7.05)	<0.001	7.28(7.2-7.33)	7.35(7.28-7.44)	0.039
**PCO**_**2**_** (mmHg)**	38(34-47)	38.5(34-48.3)	38(30-41.5)	0.540	38(32.3-45.2)	42(34.5-49)	0.274
**HCO**_**3**_** (mmol/L)**	18.5±4.6	19.2±4.2	11.4±3.0	<0.001	17.6±4.4	21.9±4	0.003
**Lactate (mmol/L)**	3.7(1.8-5.2)	3.2(1.5-4.8)	6.2(3.9-7.9)	0.010	4.1(2-6.6)	2(1.1-2.9)	0.003
**Blood urea nitrogen (mg/dL)**	50(30.9-96.8)	47(28-92.9)	74.5(36-102)	0.235	40(28-65.4)	119.5(89.5-164.3)	<0.001
**Serum creatinine (mg/dL)**	2(1.4-3.2)	2(1.2-3)	2.6(1.8-4.7)	0.166	2(1.4-2.9)	2.6(0.8-4.7)	0.409
**Serum sodium (mmol/L)**	141.9±9.5	141.7±9.6	143±9.3	0.729	138.5±7.1	153.6±7.1	<0.001
**Serum potasium (mmol/L)**	5.2±1.0	5.2±1.1	5.4±0.5	0.312	5.3±1.1	4.9±0.8	0.378
**Serum albumin (g/L)**	24.5±6.3	25.5±6	17.9±2.9	0.001	25.1±6.9	22.5±2.8	0.241
**Serum calcium (mg/dL)**	8.9(8.2-9.2)	8.9(7.9-9.2)	8.8(8.3-9.4)	0.842	8.7(7.8-9.2)	9.1(8.7-9.4)	0.045
**Serum phosphate (mg/dL)**	6.7±2.9	6.1±2.6	10.5±2.1	<0.001	6.8±2.9	6.1±2.7	0.413
**Creatine kinase (U/L)**	7949(562.5-38309.5)	7949(562.5-38137.5)	6652(535.25-66208)	0.801	3205.5(509.5-41140.8)	11583.5(5736.8-21243.5)	0.195
**Myoglobin (ng/mL)**	4007(2488.5-4007)	4007(2554.8-4007)	4003(2282.3-4007)	0.785	4003.5(2360.3-4007)	4007(2583.3-4007)	0.214
**AST (U/L)**	247.5(132-926.5)	239.5(129.8-926.5)	280(208.8-995)	0.413	249.5(92.3-1015.8)	229(132-292)	0.359
**ALT (U/L)**	153.5(75-304.8)	153.5(76.8-315.5)	165(75-305.3)	0.925	160(68.5-411.5)	153.5(107.8-172.8)	0.814
**Lactate dehydrogenase (U/L)**	732(343.8-1043.5)	732(332-1043.5)	712(407.5-2161)	0.529	552(285-1125.3)	836.5(549-982.8)	0.229
**Uric acide (mg/dL)**	10.3(7.1-14)	9.9(6.9-13.6)	12.6(9.6-14)	0.099	9.9(6.8-13.9)	11(7.6-14.8)	0.228
**C-reactive protein (mg/L)**	135.5(75.8-193)	129(74.8-193)	162(91.8-238)	0.339	141.5(79.8-212.5)	111.5(62.9-169.5)	0.350
**Procalcitonin (ng/mL)**	9.4(2.4-25.2)	8.5(2.2-25.2)	11.6(3.1-25)	0.667	11.6(3.1-26.8)	3.2(1.15-6.6)	0.010
**INR**	1.2(1-1.6)	1.2(1-1.5)	2.2(1.5-3.5)	0.002	1.2(1-1.7)	1.3(1.2-1.5)	0.281
**aPTT (sn)**	30(24.4-36.5)	28.5(23.9-35.3)	35.5(33.1-42.5)	0.017	31.5(25.5-38)	21.8(18.8-33.1)	0.025
**Fibrinogen (mg/dL)**	499.1±206.1	510.5±206.9	412.9±191.9	0.242	496±219	510±149	0.799
**D-dimer (mg/L)**	9.8(5-20)	9.8(5-20.2)	10.3(4.9-30.5)	0.812	9(5-15)	17.5(6.3-21.3)	0.220
**Urine pH**	5.5(5-5.85)	5.5(5-5.925)	5(4.75-5.5)	0.054	5.5(5-6)	5(5-5.5)	0.437
**Urine density**	1018.3±10.6	1018.2±10.6	1019.6±11.9	0.777	1018±11.7	1016.9±7.1	0.599

*PCO*_*2*_ Partial pressure of carbon dioxide, *HCO*_*3*_ Bicarbonate, *AST* Aspartate aminotransferase, *ALT* Alanine aminotransferase, *INR* International normalized ratio, *aPTT* Activated partial thromboplastin time

As shown in Table 4, the limb (75.8%) was found to be the most common location of trauma in patients admitted to the ICU, followed by the thorax (46.3%), abdomen (33.9%), and head (32.3%). Compartment syndrome was observed in 38.7% and fractures in 61.3% of the patients. There was a weak negative correlation between limb trauma and age (r=-0.297, p=0.029). There was no correlation between limb injury and the development of crush syndrome and AKI or CK levels. However, there was a weak positive correlation between limb trauma and RRT (*r*=0.256, *p*=0.045). There was a moderately positive correlation between limb trauma and the transfusion of packed red blood cells (*r*=0.335, *p*=0.008). Pneumothorax was observed in 24.2% of the patients, necessitating tube thoracostomy.

**Table 4:** Trauma findings and associated complications for earthquake victims

**Characteristics**		**7-day mortality**			**Duration under the rubble**		
	**All patients (*n*=62)**	**Survivor (*n*=54)**	**Nonsurvivor (*n*=8)**	***p*-value**	**≤72 hours (*n*=48)**	**>72 hours (*n*=14)**	***p*- value**
**n (%)**							
**Trauma locations**							
Limb	47(75.8)	42(77.8)	5(62.5)	0.388	35(74.5)	12(85.7)	0.484
Thorax	25(46.3)	21(38.9)	4(50)	0.703	19(39.6)	6(42.9)	0.826
Abdominal	21(33.9)	18(33.3)	3(37.5)	1.000	18(37.5)	3(21.4)	0.346
Head	20(32.3)	18(33.3)	2(25)		14(29.2)	6(42.9)	0.349
Pelvic	12(19.4)	11(20.4)	1(12.5)		8(16.7)	4(28.6)	0.442
Spinal	16(25.8)	14(25.9)	2(25)		11(22.9)	5(35.7)	0.488
**Thorax**							
Pneumothorax	15(24.2)	12(22.2)	3(37.5)		10(20.8)	5(35.7)	0.296
Right	2(3.2)	2(3.7)			1(2.1)	1(7.1)	
Left	7(11.3)	6(11.1)	1(12.5)		5(10.4)	2(14.3)	
Bilateral	6(9.7)	4(7.4)	2(25)		4(8.3)	2(14.3)	
Hemothorax	7(11.3)	6(11.1)	1(12.5)		5(10.4)	2(14.3)	0.651
Pneumomediastinum	10(16.1)	9(16.7)	1(12.5)		7(14.6)	3(21.4)	0.681
Subcutaneous emphysema	9(14.5)	8(14.8)	1(12.5)		7(14.6)	2(14.3)	1.000
Lung contusion	14(22.6)	10(18.5)	4(50)	0.069	9(18.8)	5(35.7)	0.274
**Compartment syndrome**	24(38.7)	22(40.7)	2(25)		18(37.5)	6(42.9)	0.717
**Fracture**	38(61.3)	34(63)	4(50)	0.700	29(60.4)	9(64.3)	0.794
**Crush syndrome**	60(96.8)	52(96.3)	8(100)	1.000	46(95.8)	14(100)	1.000
**Acute kidney injury**	55(88.7)	47(87)	8(100)	0.580	44(91.7)	11(78.6)	0.184
**Oliguria/anuria**	20(32.3)	15(27.8)	5(62.5)	0.098	18(37.5)	2(14.3)	0.192
**Deep venous thrombosis**	1(1.6)	1(1.9)			1(2.1)		
**Trauma-induced coagulopathy**	2(3.2)	2(3.7)			2(4.2)		
**Infection**	54(87.1)	47(87)	7(87.5)	1.000	41(85.4)	13(92.9)	0.670
**Sepsis**	54(87.1)	47(87)	7(87.5)	1.000	41(85.4)	13(92.9)	0.670
**Septic shock**	22(35.5)	15(27.8)	7(87.5)	0.002	16(33.3)	6(42.9)	0.539

*ICU* Intensive care unit

As presented in Table 4, of the patients, 87.1% had an infection, 87.1% had sepsis, and 35.5% had septic shock. Oliguria or anuria was present in 32.3% of the patients. Acute kidney injury developed in 55 (88.7%) of the patients, of whom 20 (36.4%) had oliguria or anuria, 36 (65.5%) had hyperpotassemia, and 38 (69.1%) patients had an indication for RRT. There was a positive correlation between the development of AKI and myoglobin, serum lactate, and uric acid levels (*r* = 0.372, *p* = 0.003; *r* = 0.307, *p* = 0.016; *r* = 0.428, *p* = 0.001, respectively). Crush syndrome developed in 96.8% of the patients. Of the patients with crush syndrome, 54 (90%) had AKI, 20 (33.3%) had oliguria or anuria, and 40 (66.7%) had an indication for RRT. A moderately positive correlation was observed between the presence of crush syndrome and infection and sepsis (*r* = 0.443, *p*<0.001; *r* = 0.474, *p*<0.001). Two patients had inhalation injury on admission to the ICU. One patient with trauma in all four extremities developed a fat embolism during ICU follow-up. Abdominal compartment syndrome developed in two patients (Table 5). Small bowel necrosis developed in one of these patients, and segmental resection of the small bowel was performed. The other patient underwent emergency decompression surgery and was followed up as an open abdomen.

**Table 5:** Clinical characteristics of patients with high intra-abdominal pressure during admission to the intensive care unit

**Patients**	**APACHE II score**	**Abdominal trauma**	**Crush syndrome**	**MAP (mmHg)**	**IAP (mmHg)**	**APP (mmHg)**	**AKI**	**Oliguria**	**RRT**	**Furosemide**	**NTG**	**Human serum albumine**	**IMV**	**Mortality**
1	8	Yes	Yes	125	10.3	114.7	Yes	Yes	Yes	Yes	Yes	No	No	No
2	15	No	Yes	100	11	89	Yes	No	No	Yes	No	Yes	No	No
3	38	No	Yes	88	8.1	79.9	Yes	No	Yes	Yes	No	Yes	Yes	No
4	12	Yes	Yes	90	11	79	No	No	No	No	No	No	No	No
5*	20	Yes	Yes	90	9.6	80.4	Yes	Yes	Yes	No	No	Yes	No	No
6	47	No	Yes	62	11	51	Yes	No	Yes	Yes	No	Yes	Yes	No
7	14	No	Yes	87	10.3	76.7	Yes	No	Yes	Yes	Yes	Yes	No	No
8	4	Yes	Yes	108	11.8	96.2	Yes	No	No	Yes	Yes	No	Yes	No
9	49	No	Yes	60	10.3	49.7	Yes	No	Yes	Yes	No	Yes	Yes	No
10	26	No	Yes	94	7.4	86.6	Yes	No	Yes	Yes	No	Yes	No	No
11	18	Yes	Yes	90	10.3	79.7	Yes	No	Yes	Yes	No	Yes	Yes	No
12	20	Yes	Yes	60	11	49	Yes	Yes	Yes	Yes	Yes	Yes	Yes	No
13	8	No	Yes	66	8.8	57.2	Yes	Yes	Yes	Yes	Yes	No	No	No
14	32	No	Yes	72	8.8	63.2	Yes	Yes	Yes	Yes	Yes	Yes	No	No
15	10	Yes	Yes	90	8.8	81.2	Yes	No	Yes	Yes	No	yes	No	No
16	37	No	Yes	81	10.3	70.7	Yes	Yes	Yes	Yes	No	Yes	Yes	Yes
17**	29	Yes	Yes	88	7.4	80.6	Yes	Yes	Yes	Yes	No	Yes	Yes	No
18	34	No	Yes	76	7.4	68.6	Yes	No	No	Yes	No	Yes	Yes	No

*APACHE* Acute Physiology and Chronic Health Evaluation, *MAP* Mean arterial pressure, *IAP* Intraabdominal pressure, *APP* Abdominal perfusion pressure, *AKI* Acute kidney injury, *RRT* Renal replacement therapy, *NTG* Intravenous nitroglycerin, *IMV* Invasive mechanical ventilation

^*^ The patient underwent emergency decompression surgery and was followed up as an open abdomen

^**^ Small bowel necrosis developed in this patient and segmental resection of the small bowel was performed

Table 6 demonstrates the treatments and clinical courses for earthquake victims in the ICU. Twenty-six (41.9%) patients needed invasive mechanical ventilation (MV) during ICU admission. More than half of the patients required a blood transfusion. Renal replacement therapy was performed in 40 (64.5%) patients. Trauma-associated operations were performed in 56.5% of the patients, amputation in 41.9%, and fasciotomies in 21%. Amputation was performed in 26 (55.3%) patients with limb trauma.

**Table 6:** Treatments and clinical courses for earthquake victims

**Characteristics**		**7-day mortality**			**Duration under the rubble**		
	**All patients (n=62)**	**Survivor (*n*=54)**	**Nonsurvivor (*n*=8)**	***p*-value**	**≤72 hours (*n*=48)**	**>72 hours (*n*=14)**	***p*- value**
**n (%) or median (Q1-Q3)**							
**Isotonic saline**	62(100)	54(100)	8(100)		48(100)	14(100)	
**5% dextrose**	46(74.2)	40(74.1)	6(75)	1.000	34(70.8)	12(85.7)	0.322
**Human serum albumin**	37(59.7)	36(66.7)	1(12.5)		27(56.3)	10(71.4)	0.308
**Blood transfusions**	45(72.6)	42(77.8)	3(37.5)		36(75)	9(64.3)	0.502
Packed red blood cells	42(67.7)	40(74.1)	2(25)		34(70.8)	8(57.1)	0.349
Platelet transfusions	9(14.5)	9(16.7)			8(16.7)	1(7.1)	
Fresh frosen plasma	7(11.3)	6(11.1)	1(12.5)		5(10.4)	2(14.3)	
Cryoprecipitate	1(1.6)		1(12.5)		1(2.1)		
**Fluid input**	4.8(3.1-5.8)	4.8(3-5.7)	5.6(4.5-9)	0.143	4.7(2.9-5.6)	4.8(3.7-6.4)	0.370
**Urine output**	1.8(0.3-2.6)	1.8(0.4-2.9)	0.4(0.2-1.9)	0.053	1.8(0.2-2.6)	1.9(1.9-3.1)	0.220
**Renal replacement therapy**	40(64.5)	38(70.4)	2(25)	0.019	33(68.8)	7(50)	0.219
**Furosemide**	44(71)	43(79.6)	1(12.5)		35(72.9)	9(64.3)	0.524
**Intravenous nitroglycerin**	14(22.6)	14(25.9)			11(22.9)	3(21.4)	1.000
**Vasopressor**	24(38.7)	16(29.6)	8(100)	<0.001	17(35.4)	7(50)	0.324
**Inotropic agent**	12(19.4)	8(14.8)	4(50)	0.039	7(14.6)	5(35.7)	0.121
**Anticoagulant therapy**	38(61.3)	36(66.7)	2(25)		31(64.5)	7(50)	0.324
**Sedation**	30(48.4)	22(40.7)	8(100)	0.002	23(47.9)	7(50)	0.891
**Analgesia**	61(98.4)	53(98.1)	8(100)	1.000	47(97.9)	14(100)	1.000
**Sympathetic nerve blocks**	3(4.8)	1(1.9)	2(25)		3(6.3)		
**Oxygen therapy on ICU admission**							
No	9(14.5)	9(16.7)			6(12.5)	3(21.4)	
Nasal cannula	18(29)	18(33.3)			15(31.3)	3(21.4)	
Mask with small diffuser	3(4.8)	3(5.6)			3(6.3)		
Nonrebreathing mask	5(8.1)	5(9.3)			5(10.4)		
High flow nasal cannula oxygen	1(1.6)	1(1.9)			1(2.1)		
Invasive mechanical ventilation	26(41.9)	18(33.3)	8(100)		18(37.5)	8(57.1)	
**Oxygen therapy during ICU**							
High flow nasal cannula oxygen	15(24.2)	14(25.9)	1(12.5)		14(29.2)	1(7.1)	
Noninvasive mechanical ventilation	12(19.4)	9(16.7)	3(37.5)		7(14.6)	5(35.7)	0.121
Invasive mechanical ventilation	26(41.9)	20(37)	6(75)	0.059	18(35.7)	8(57.1)	0.190
**Tube thoracostomy**	15(24.2)	12(22.2)	3(37.5)		9(18.8)	6(42.9)	0.082
**Fasciotomy**	13(21)	12(22.2)	1(12.5)		10(20.8)	3(24.4)	
**Amputation**	26(41.9)	26(48.1)			16(33.3)	10(71.4)	0.011
**Trauma associated operation**	35(56.5)	34(65)	1(12.5)		26(54.2)	9(64.3)	0.502
**ICU length of stay (days)**	5.5(2-1)	7(3.8-11)	1.5(1-2)	<0.001	5.5(2-10.8)	5(2.8-9)	0.776
**Hospital length of stay (days)**	11(7.8-14)	11(9-14)	1.5(1-2)	<0.001	11(8-14)	9(6.8-11)	0.074
**7-day mortality**	8(12.9)				6(12.5)	2(14.3)	

*ICU* Intensive care unit

The 7-day mortality rate was 12.9%. All of the nonsurvivors had crush syndrome and AKI. According to mortality, in univariate analysis, parameters affecting survival were evaluated with logistic regression analysis. There was no factor affecting survival according to multivariate analysis.

**Fig. 2:**
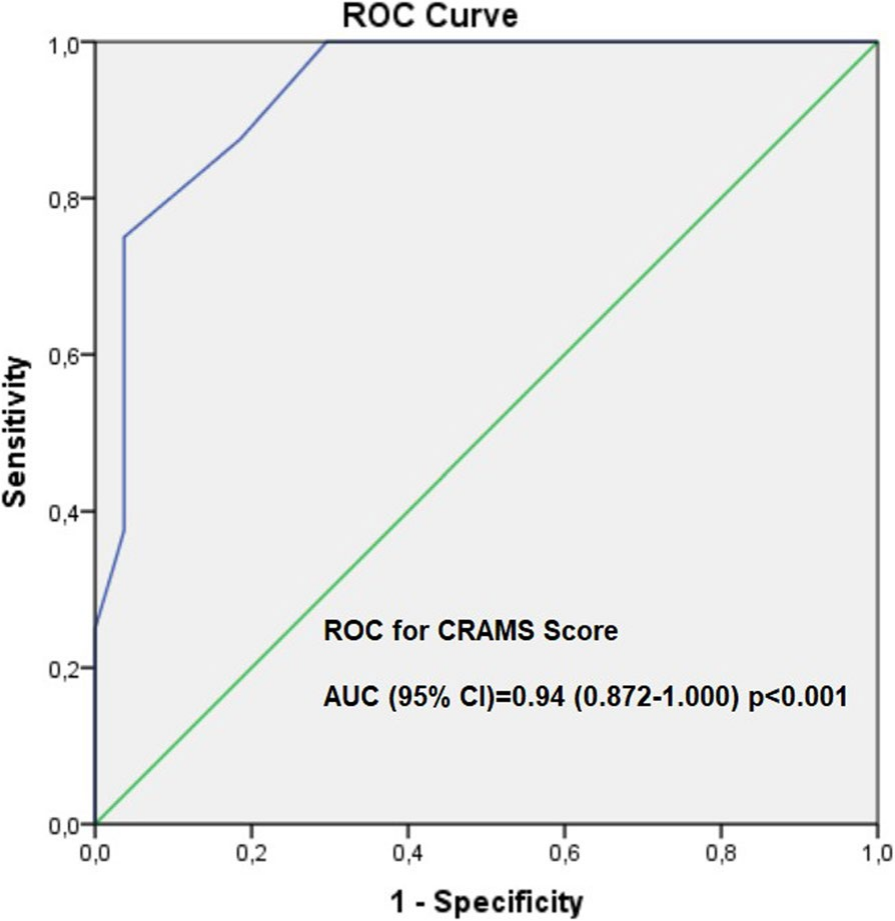
ROC analysis of the CRAMS score in order to predict 7-day mortality

A ROC analysis was performed to determine the diagnostic accuracy of the CRAMS score (Fig. 2). The best cutoff of the CRAMS score to predict in-7-day mortality was < 4.5 with 0.94 area under the curve (AUC); application of this threshold resulted in 75% sensitivity and 96.3% specificity (*p*<0.001). The Youden index was 0.713.

## Discussion

These earthquakes that occurred in our country have been labeled the 'disaster of the century', which is unprecedented in history in terms of severity and area covered. Thousands of people were left under the rubble. Our hospital was also damaged after the earthquake’s epicenter in Yayladagi on February 20, 2023, and our patients had to be evacuated immediately. Earthquakes have happened thus far in the world and in our country and unfortunately will continue to occur. In this study, we aimed to guide clinicians in future earthquakes by sharing our experiences in the disaster of the century.

The majority of the earthquake victims in the study came from Hatay Province, one of the cities particularly affected by the earthquake. As in the study by Koyuncu et al., the earthquake victims in this study were young [10]. Comorbidities in elderly patients may reduce the survival rate under the rubble. We observed that the earthquake victims who died remained under the rubble for a longer period of time. This period was longer than that in previous studies [10, 11]. However, there was no statistically significant difference between the duration of being under the rubble and mortality. While hypernatremia developed in the patient, who was under the rubble for a maximum of 222 hours, no AKI was observed, and the patient was transferred to the service. Based on this finding and a previous study, search and rescue efforts should be continued for at least ten days, and it should not be forgotten that people may still be alive under the rubble [2].

To determine the prognosis of trauma and ICU patients, many scoring systems are used [2, 3, 5, 6]. It was observed that the APACHE II score was statistically higher in patients who spent more than 72 hours under the rubble. Mortality rates were 12.5% and 14.3% for those who were trapped under the rubble for 72 hours or less and for longer than 72 hours, respectively. Studies have shown that the CRAMS score is one of the best predictors of trauma severity, as in this study [5, 12, 13]. In a recent study, RTS and CRAMS scores were shown to predict mortality better than the Early Warning Score in high-energy trauma patients [5]. Looking at the literature, there are few studies of the CRAMS score in earthquake victims [12–15]. He et al. found that it can be used for rapid assessment of trauma severity in earthquake victims [12]. Tang et al. showed that the CRAMS score gave consistent results in assessing the injury status of individuals [14]. In this study, it was observed that a low CRAMS score was associated with the development of AKI and crush syndrome. In other words, the more severe the trauma, the more damage-related crush syndrome and one of its complications, AKI, can develop. Considering the results of our study and previous studies, the CRAMS score is a scoring system that can be used to evaluate trauma severity and complications related to earthquakes [5, 12–14].

Crush injuries occur as a result of direct trauma to the tissue and compression of the body in one place [2]. Edema resulting from bleeding and tissue damage may increase intracompartmental pressure, which can cause the development of compartment syndrome [16]. Compartment syndrome was observed in 38.7% of our patients. Increased intracompartmental pressure causes hypoperfusion, resulting in tissue hypoxia and ischemi. This causes mitochondrial dysfunction and, ultimately, cellular damage. As a result of the destruction of the damaged muscle, intracellular components enter the systemic circulation. As in this study, potassium, phosphorus, uric acid, myoglobin, CK, lactate dehydrogenase, and aspartate aminotransferase levels increased in the blood [2, 11, 17]. Injured cells can also cause platelet aggregation and vasoconstriction. Inflammatory mediators are released, increasing vascular permeability. Hypovolemia and shock may develop as a result of fluid passage into the intracellular space, low or no fluid intake as a result of being under rubble, or bleeding due to trauma [2]. It is therefore very important to begin fluid therapy for victims as soon as their limbs are seen at the scene. If adequate fluid therapy could not be given beforehand to those rescued from the rubble, damage would occur as a result of the reperfusion of hypoperfused tissues. Crush syndrome is seen in approximately 40–70% of patients with crush injuries [2]. This rate was higher in this study compared to the literature. This result may have resulted from the long duration of being under the rubble due to the consequences of the severity of the earthquake. Crush syndrome is an ischemia‒reperfusion injury. Systemic findings occur as a result of damage, and organ dysfunction can be seen. As also found in our study, these systemic manifestations are bleeding, hypovolemic shock, metabolic disorders, AKI, acute respiratory distress syndrome, sepsis, arrhythmia, disseminated intravascular coagulation, and psychological trauma [2, 11, 17]. Consumption coagulopathy may occur as a result of trauma, systemic inflammation, and increased tissue thromboplastin levels.

Many metabolic disorders can be seen in earthquake victims. Patients may develop metabolic acidosis due to cellular necrosis, shock, uremia, and lactic acid secretion. Lactate was higher and pH and bicarbonate levels were lower in the group of patients who stayed under the rubble for a shorter time. Although not statistically significant in our results, AKI, need for RRT and blood transfusion rates were higher in earthquake victims who stayed under the rubble for a shorter time. Patients may have developed hypoperfusion, lactic acidosis and AKI due to hemorrhage. Lactic acidosis, AKI and the need for RRT may have developed as a result of ischemia–reperfusion injury due to inadequate fluid replacement at the time of rescue in patients rescued in the early period. While lactate was significantly higher in nonsurvivors, pH and bicarbonate were lower. Lactic acidosis may have developed as a result of the anaerobic glycolysis that occurred due to the high severity of trauma, the high rate of organ dysfunction, and the high rate of septic shock in the nonsurvivors. Severe acidosis can reduce myocardial contractility and cardiac output, and patients may require hemodialysis. Hyponatremia, thought to be due to many pathophysiologies, has been observed in previous studies [11, 18]. In this study, the mean serum sodium level of all patients was found to be within the normal range. Unlike other studies, hypernatremia was observed in earthquake victims who remained under rubble for more than 72 hours. This was different from Zhang et al.'s study and suggests that our patients had hypovolemic hypernatremia because the duration of their stay under the rubble was longer than that in previous earthquake studies [18].

The severity of crush-related AKI depends on the extent of muscle damage, the degree of hypovolemia, underlying comorbidities, and the development of complications. Insufficient fluid intake or trauma-related hemorrhage due to prolonged exposure of victims to rubble may result in decreased intravascular volume. In salvage, there is volume loss in the intravascular space as a result of fluid leaking into the reperfused interstitial space. For these reasons, patients may develop prerenal AKI. In the current study, there was a significant correlation between lactate level, which is a global perfusion indicator, and the development of AKI. AKI may also develop as a result of myoglobin, uric acid, and phosphorus, which occur as a result of tissue damage, causing tubular damage, as shown in this study [10, 17, 19].

Fluid management is very important in these patients, and both hypovolemia and hypervolemia are harmful. As most patients are hypovolemic, loop diuretics may not be beneficial or may even be harmful in AKI [2]. If the patient is hypervolemic, loop diuretics may be tried [2]. With the technological developments in ICUs, ultrasound, which is described as the new stethoscope of ICUs, has made it easier for us to perform our fluid management correctly. The rates of loop diuretics, intravenous nitroglycerin administration, and the need for RRT were high in survivors. How much fluid replacement is important during recovery, while giving more fluid than needed afterwards may also contribute to the development of compartment syndrome. For these reasons, correct fluid management is lifesaving for critical earthquake victims. Intra-abdominal pressure monitoring should be performed in patients with suspected increased intra-abdominal pressure, as in our study. The low diameter of the inferior vena cava may be misleading in this patient group, and other methods should be used to help evaluate the fluid status.

As in this study, the most commonly injured areas are the limbs and thorax [2]. A positive correlation was observed between limb trauma and blood transfusion. This may have been due to blood loss due to vascular damage caused by limb injury. Patients should be closely monitored for compartment syndrome (6P sign). Early diagnosis and treatment reduces the risk of compartment syndrome and limb loss. In the event of compartment syndrome, a fasciotomy is performed, and amputation may be necessary in some patients [16]. As a result of trauma, lung contusion, pneumothorax, hemothorax, pneumomediastinum, heart contusion, rib fracture, pelvic fracture, liver injury, spleen injury, and spinal cord damage can be seen in patients. Inhalation injury may occur as a result of the inhalation of gases released at the site of the rubble, as seen in two of our patients [2]. As in one of our patients, a fat embolism can be observed, especially after long bone fractures. Invasive mechanical ventilation support may be required in patients who develop acute respiratory failure as a result of trauma-related pulmonary events.

Tissue damage results in damage to the protective barrier of the skin. Open wounds may become infected as a result of the patient being under the rubble for a long time [2, 20, 21]. Causes such as fasciotomies or having to perform amputations at the site of rubble may cause contamination and infection. A wound infection may require debridement. Yalin and Golgelioglu showed that the number of fasciotomy incisions in earthquake patients who acquired sepsis after the Kahramanmaras earthquake was statistically considerably greater than in those who did not develop sepsis [17]. The rates of infection and sepsis were quite high in our study. There was also a positive correlation between infection and sepsis in earthquake victims who developed crush syndrome. However, we did not observe a significant difference between the duration of the stay under rubble and the rate of infection and sepsis. This may have resulted from the removal of damaged tissues, as the amputation rate was higher in patients who had been under rubble for a long time. In their study, Keven et al. showed that the mortality rate was high in patients with infection and sepsis [22]. In our study, the rate of septic shock was higher in nonsurvivors. While amputation of severely crushed limbs saves the patient's life (3-58%), delays in fasciotomies may lead to limb loss [2, 16].

Mortality was observed only in patients from Hatay in this study. In Hatay, a significant number of health institutions were rendered unusable as a result of earthquake-related damage, and a significant number of health workers died or were injured. In previous studies, while the mortality rate in crush syndrome was 20%, it was higher in multiorgan failure [2]. The SOFA score, which is used as an indicator of organ dysfunction, and the mNUTRIC score, which indicates disease severity and malnutrition, were found to be significantly higher in the nonsurvivors. In this study, the7-day mortality rate was 12.9%. We could not evaluate the 28-day mortality because our hospital was damaged after the Hatay earthquake of February 20, 2023, and we had to evacuate the hospital. We think that our mortality is low as a result of correct fluid management by dynamic measurements.

One of the strengths of this study is that it deals in detail with the clinical characteristics and management of critical earthquake survivors after the catastrophic earthquakes of the century and has real-life data. Second, when the literature was analyzed, while the experiences at the scene were mostly shared, no study was observed in which the patient data in the ICU were discussed in such detail. Of course, this study has some limitations. First, the single-center and retrospective nature of the study may have affected its quality. Second, we could not evaluate 28-day mortality because our hospital was evacuated during the second major earthquake.

## Conclusions

The CRAMS score can be used to assess the severity of trauma and predict mortality in critically ill earthquake victims. The severity of the trauma affects the survival of the victim more than the duration of being under the rubble. For this reason, search and rescue operations should continue for at least one week and ten days after an earthquake. The most commonly injured areas among earthquake victims were the limbs. Hypernatremia and high BUN levels secondary to hypovolemia, high CK levels, and higher amputation rates due to crush injuries have been observed in earthquake victims who have been under the rubble for more than 72 hours. AKI can develop due to myoglobin, lactate and uric acid released from the cell into the circulation as a result of muscle crush injury. Infection, sepsis, and septic shock were observed at high rates in earthquake victims as a result of tissue damage. It is vital to start treating earthquake victims with fluids low in potassium as soon as limbs are seen at the scene. This may prevent the development of ischemia-reperfusion injury, the development of crush syndrome, and its complications. For these reasons, management of crush syndrome and complications is life-saving. Performing fasciotomies and amputations at the right time can reduce morbidity and mortality. If there is a suspicion of increased intra-abdominal pressure in earthquake victims, monitoring of intra-abdominal pressure may reduce the complications that may occur. Both hypovolemia and hypervolemia can cause dangerous consequences for critically ill earthquake victims. Normovolemia should be ensured by hemodynamic monitoring with dynamic measurements.

## Data Availability

The datasets generated and/or analyzed during the current study are not publicly available as they contain patient information, but the data that supports the findings of this study is available from the corresponding author (KA) on reasonable request.

## Abbreviations

AKI: Acute kidney injury
AUC: Area under the curve
APACHE: Acute physiology and chronic health evaluation
BUN: Blood urea nitrogen
CCI: Charlson comorbidity index
CK: Creatinine kinase
CRAMS: Circulation, Respiration, Abdomen, Motor, and Speech
GCS: Glasgow coma scale
ICU: Intensive care unit
ISS: Injury severity score
mNUTRIC: Modified nutrition risk in the critically ill
MV: Mechanical ventilation
ROC: Receiver operating characteristic
RRT: Renal replacement therapy
RTS: Revised trauma score
SOFA: Sequential organ failure assessment
SpO_2_: Peripheral arterial oxygen saturation

## References

[1] 2023 Kahramanmaras and Hatay earthquake report. Available from: 2023 Kahramanmaraş ve Hatay Depremleri Raporu (sbb.gov.tr). Accessed 3 Sept 2023.

[2] Long B, Liang SY, Gottlieb M (2023). Crush injury and syndrome: a review for emergency clinicians. Am J Emerg Med.

[3] Li W, Qian J, Liu X, Zhang Q, Wang L, Chen D (2009). Management of severe crush injury in a front-line tent ICU after 2008 Wenchuan earthquake in China: an experience with 32 cases. Crit Care.

[4] Huang X, Guo Q, Li C, Wang Y, Song L, Shang K (2014). Intensive care unit treatment strategy and algorithm for critical patients from Lushan earthquake. Zhonghua Yi Xue Za Zhi.

[5] Yolcu S, Sener K, Tapsiz H, Ozer AI, Avci A (2023). Revised Trauma Score and CRAMS better predicted mortality in high-energy-trauma patients than Early-Warning Score. Ir J Med Sci.

[6] Yang YW, Wu CH, Tsai HT, Chen YR, Chang YP, Han YY (2023). Dynamics of immune responses are inconsistent when trauma patients are grouped by injury severity score and clinical outcomes. Sci Rep.

[7] Quintairos A, Pilcher D, Salluh JIF (2023). ICU scoring systems. Intensive Care Med.

[8] Mahmoodpoor A, Sanaie S, Sarfaraz T, Shadvar K, Fattahi V, Hamishekar H (2023). Prognostic values of modified NUTRIC score to assess outcomes in critically ill patients admitted to the intensive care units: prospective observational study. BMC Anesthesiol.

[9] Rajasurya V, Surani S (2020). Abdominal compartment syndrome: often overlooked conditions in medical intensive care units. World J Gastroenterol.

[10] Koyuncu S, Sipahioglu H, Bol O, Ilik HKZ, Dilci A, Elmaagac M (2023). The evaluation of different treatment approaches in patients with earthquake-related crush syndrome. Cureus.

[11] Safari S, Eshaghzade M, Najafi I, Baratloo A, Hashemi B, Forouzanfar MM (2017). Trends of serum electrolyte changes in crush syndrome patients of bam earthquake; a cross sectional study. Emerg (Tehran).

[12] He Y, Hu H, Jiang Y, Hu J, Li X, Yao Y (2014). Comparison of the performance of three prehospital trauma scores in evaluation of injury severity among Lushan earthquake victims. Zhonghua Wei Zhong Bing Ji Jiu Yi Xue.

[13] Jiang X, Jiang P, Mao Y (2019). Performance of Modified Early Warning Score (MEWS) and Circulation, Respiration, Abdomen, Motor, and Speech (CRAMS) score in trauma severity and in-hospital mortality prediction in multiple trauma patients: a comparison study. PeerJ.

[14] Tang S, Ni F, Hu H, Du X, Zhu S, Wang H, Niu Z, He Y, Cao Y (2022). Injury assessment of individuals wounded in the lushan earthquake and the emergency department workload: a corresponding correlation study. Disaster Med Public Health Prep.

[15] He YR, Hu H, Jiang YW, Hu JF, Li XH, Yao YC (2014). Multivariate factors analysis on length of stay in Lushan earthquake victims. Sichuan Da Xue Xue Bao Yi Xue Ban..

[16] Kundakci B, Mirioglu A, Tekin M, Bagir M, Bicer OS, Arslan YK (2023). 6 February 2023, orthopedic experience in Kahramanmaraş earthquake and surgical decision in patients with crush syndrome. J Orthop Surg Res.

[17] Yalın M, Gölgelioğlu F (2023). A Comparative Analysis of Fasciotomy Results in Children and Adults Affected by Crush-Induced Acute Kidney Injury following the Kahramanmaraş Earthquakes. Medicina (Kaunas).

[18] Zhang L, Fu P, Wang L, Cai G, Zhang L, Chen D (2013). Wenchuan earthquake-related AKI study group. Hyponatraemia in patients with crush syndrome during the Wenchuan earthquake. Emerg Med J.

[19] Sever MS, Luyckx V, Tonelli M, Kazancioglu R, Rodgers D, Gallego D (2023). Disasters and kidney care: pitfalls and solutions. Nat Rev Nephrol.

[20] Ates S, Erdem H (2023). The earthquake in Türkiye and infectious disease concerns. New Microbes New Infect.

[21] Ulusoy S, Kılınc İ, Oruc M, Ozdemir B, Ergani HM, Keskin OH (2023). Analysis of wound types and wound care methods after the 2023 Kahramanmaras earthquake. Jt Dis Relat Surg.

[22] Keven K, Ates K, Sever MS, Yenicesu M, Canbakan B, Arinsoy T (2003). Infectious complications after mass disasters: the Marmara earthquake experience. Scand J Infect Dis.

